# Nationwide analysis of surgical and interventional uterine fibroid treatments over the past decades in Germany

**DOI:** 10.1186/s42155-025-00578-3

**Published:** 2025-08-04

**Authors:** Katharina Rippel, Josua Decker, Thomas Kroencke, Christian Scheurig-Muenkler

**Affiliations:** 1 Diagnostic and Interventional Radiology, Faculty of Medicine, University Hospital Augsburg, University of Augsburg, Stenglinstr. 2, Augsburg, 86156 Germany; 2 Centre for Advanced Analytics and Predictive Sciences (CAAPS), University of Augsburg, Universitätsstr. 2, Augsburg, 86159 Germany

**Keywords:** Uterine artery embolization, Hysterectomy, Myomectomy, Uterine fibroids

## Abstract

**Background:**

Uterine artery embolization (UAE) has been a valid tool in the treatment of symptomatic uterine fibroids for a quarter of a century by now. This study aims to examine the impact of this treatment and its development since its introduction in Germany.

**Materials and methods:**

All patients that received inpatient treatment for uterine fibroids (ICD-10 code D25) as primary diagnosis or the primary symptoms of hypermenorrhea/menorrhagia (N92) or dysmenorrhea (N94) with a secondary diagnosis of uterine fibroids from 2005 until 2023 were included. The data was obtained from the research data center of the German Federal Statistical Office. Basic demographic data and all encoded diagnostic and therapeutic procedures were collected and analyzed. Special focus laid on patients receiving either of the three invasive treatments: hysterectomy (HE), myomectomy (ME), and uterine artery embolization (UAE).

**Results:**

One million one hundred sixty-four thousand five hundred sixty-six women were hospitalized in those 19 years with 91.7% receiving either HE, ME or UAE. There was a continuous decline in annual numbers of hospitalizations (-47.7%) and of HE (-55.5%) between 2005 and 2023. Constantly more than 99% of the treated women underwent surgery—contribution of UAE continuously remained below 1%. The highest number of UAE was conducted in 2012 with 568 treated women, followed by a continuous decline to 313 in 2023. The reference population of all women at the age of 30 to < 55 years in Germany decreased only slightly from 15,230,000 to 13,506,000 (-11.3%).

**Conclusion:**

The numbers of inpatient invasive treatment continuously declined disproportionately to the demographic development. Despite excellent evidence, UAE is not used for treatment of symptomatic uterine fibroids to a relevant extent in Germany.

## Background

Over the past two decades, uterine artery embolization (UAE) has been introduced, refined, and rigorously studied as a minimally invasive treatment alternative for symptomatic uterine fibroids, meeting the highest scientific standards. Numerous studies, including several randomized controlled trials, have demonstrated that UAE is at least equally effective in terms of clinical outcomes and cost-efficiency compared to standard therapies, such as hysterectomy (HE) and myomectomy (ME) [1–7]. This is accompanied by clear advantages, including minimal invasiveness and organ preservation. Even the last remaining contraindication—desire for children—which had been upheld due to a lack of study data, is beginning to diminish, among other studies based on the current results of the FEMME trial and the subsequent evaluation by its authors [8–10]. As a result of all these efforts and findings, UAE is recommended as a treatment alternative equal to HE and ME in Germany with the clear demand, that affected women should be informed about all aspects of these treatment options [11–13]. Comparable recommendations can be found internationally. This seems like a success story for interventional radiology. Nevertheless, there are international reports of underutilization of UAE, as well as indications of insufficient counseling of affected women by their treating gynecologists. As one of the early teams, we have regularly performed UAE since 2000, scientifically evaluated it and engaged in educating patients and referrers. The present analysis aims to examine the impact of this engagement and the influence of introducing UAE on the treatment of affected women in Germany.

## Material and methods

### Data source

All inpatient treatment cases in Germany are billed using the case-based payment system, which relies on defined Diagnosis Related Groups (DRGs). A broad spectrum of demographic and clinical as well as billing-relevant data related to inpatient stays are anonymized and collected in the DRG statistics of the German Federal Statistical Office. Upon request, anonymized data for scientific analysis are made available to researchers through the Research Data Center (RDC) [14]. Specific queries from the overall statistics can be processed remotely using specialized syntaxes. The earliest available accounting year is 2005. After the syntaxes written by the authors using Stata 17 were sent to the RDC, the data analysis was conducted remotely. The results were transferred after passing an anonymity check, with subgroups of fewer than five individual cases excluded. Clinical diagnoses are recorded according to the 10th version of the International Statistical Classification of Diseases and Related Health Problems classification, German modification (ICD-10-GM), while inpatient diagnostic and therapeutic procedures are coded according to the version of the OPS (“Operationen- und Prozedurenschlüssel”- diagnostic and therapeutic procedure codes) both valid for the respective accounting year. The three invasive treatment groups analysed were selected according to the following OPS codes – hysterectomy: 5–682, 5–683, 5–685; myomectomy: 5–681.2, 5–681-3 (2005–2014), 5–681.3, 5–681-8, 5–681.9 (2015–2023); uterine artery embolization: 8–836.k9 (2005–2016), 8–836.kh (2017–2023), 8–836.ka.

### Patient cohort and reference population

All completed inpatient cases during the study period from 2005 to 2023 were considered. All women with the primary or secondary diagnosis of D25 for symptomatic uterine fibroids according to ICD-10 were included. In cases where this diagnosis was recorded as a secondary diagnosis, the primary diagnosis had to reflect a clinically indicative symptomatology requiring treatment (N92 for hypermenorrhea/menorrhagia or N94 for dysmenorrhea). In addition to age and clinical diagnoses, all diagnostic and therapeutic procedures performed during the stay, as well as the length of hospitalization, were recorded. The reference population included all women in Germany at the age of 30 to under 55 years. An appropriately accessible online query tool from the Federal Statistical Office is available for this purpose (https://www-genesis.destatis.de).

### Statistical analysis

Data analyses were performed using Stata 17 and R version 4.1.2 (www.r-project.org). Categorial variables are presented with absolute numbers (n) and percentages (%). The mean with standard deviation (SD) or median with interquartile range (IQR) were used to present continuous variables as indicated.

## Results

### Baseline numbers

A total number of 1,164,566 women were included in this study. Over the 19 years’ time frame the number of inpatient cases decreased disproportionately in comparison to the reference population (*n* = 271,327,000). The absolute number of hospitalizations decreased from 82,276 in 2005 to 43,035 in 2023. This corresponds to a prevalence of 540 per 100,000 in 2005 and 319 per 100,000 in 2023 in the age group of 30 to under 55 years.

Furthermore, the number of hospitalizations without one of the three treatments fell from 7,291 in 2005 to 3,818 in 2023, which equals a constant relative percentage of 7.8 ± 0.56%. These patients received different diagnostic procedures as well as invasive and non-invasive treatments and supportive therapies.

### Treatments

**Fig. 1:**
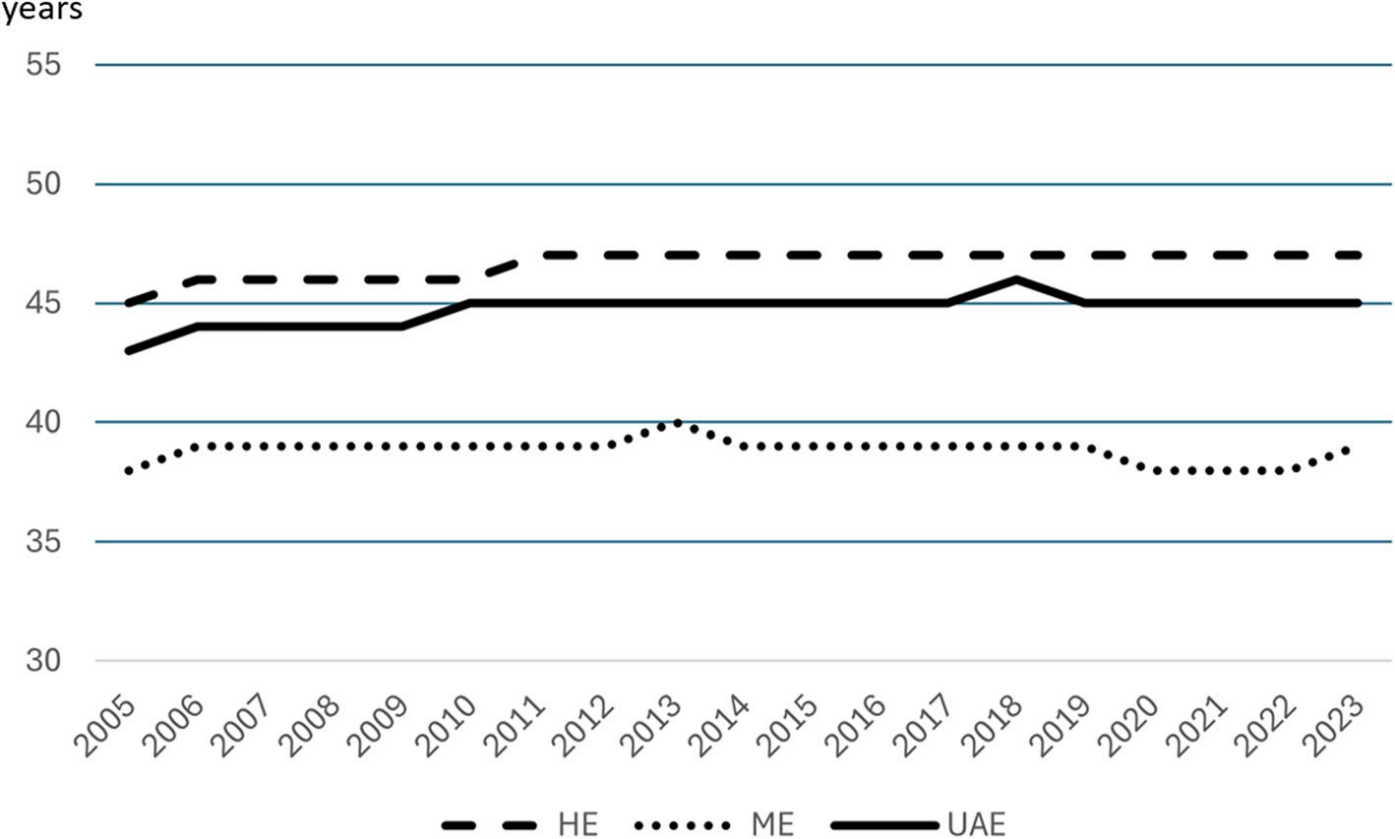
Mean age of patients being treated by UAE, HE or ME In the years from 2005–2023. UAE: uterine artery embolization, HE: hysterectomy, ME: myomectomy

Over the whole time, on average 91.8 ± 0.52% of the hospitalized patients received one of the three treatments. Thereby, the absolute numbers decreased from 74,669 in 2005 to 38,998 in 2023. Table 1 comprises the absolute numbers and percentages of the treatment strategies of all included hospitalizations from 2005 to 2023. The median age of women being treated by HE, ME and UAE was 46 (IQR: 43–50), 39 (IQR: 34–45) and 45 (IQR: 41–48) years, respectively, with no relevant fluctuation over the observed time period, as seen in Fig. 1.

**Table 1:** Absolute numbers and percentages of treatment strategies of all included hospitalizations from 2005 to 2023

Year	Reference population	Hospitalizations	Invasive treatments	Hysterectomy		Myomectomy		Uterine artery embolization		None	
	female, 30 to < 55 years	n	n	n	%	n	%	n	*%*	n	%
2005	15,230,000	82,276	74,669	64,796	*86.78*	9,598	*12.85*	275	*0.37*	7,291	*8.86*
2006	15,101,000	80,867	74,054	63,459	*85.69*	10,308	*13.92*	287	*0.39*	6,509	*8.05*
2007	14,978,000	82,109	75,378	64,630	*85.74*	10,349	*13.73*	399	*0.53*	6,374	*7.76*
2008	14,928,000	79,411	73,184	62,223	*85.02*	10,578	*14.45*	383	*0.52*	5,863	*7.38*
2009	14,818,000	76,373	70,560	59,484	*84.30*	10,663	*15.11*	413	*0.59*	5,417	*7.09*
2010	14,728,000	73,760	68,175	56,962	*83.55*	10,847	*15.91*	366	*0.54*	5,213	*7.07*
2011	14,392,000	70,580	65,362	53,927	*82.51*	11,030	*16.88*	405	*0.62*	4,828	*6.84*
2012	14,310,000	66,798	61,491	50,269	*81.75*	10,654	*17.33*	568	*0.92*	4,885	*7.31*
2013	14,251,000	62,507	57,537	46,475	*80.77*	10,587	*18.40*	475	*0.83*	4,633	*7.41*
2014	14,187,000	61,333	56,425	45,529	*80.69*	10,487	*18.59*	409	*0.72*	4,526	*7.38*
2015	14,114,000	56,801	51,901	41,363	*79.70*	10,148	*19.55*	390	*0.75*	4,619	*8.13*
2016	14,217,000	53,812	49,252	38,389	*77.94*	10,548	*21.42*	315	*0.64*	4,297	*7.99*
2017	14,067,000	51,272	46,812	36,210	*77.35*	10,203	*21.80*	399	*0.85*	4,252	*8.29*
2018	13,881,000	51,583	47,172	36,069	*76.46*	10,729	*22.74*	374	*0.79*	4,148	*8.04*
2019	13,848,000	48,756	44,497	33,658	*75.64*	10,540	*23.69*	299	*0.67*	4,036	*8.28*
2020	13,702,000	41,338	37,922	27,943	*73.69*	9,735	*25.67*	244	*0.64*	3,211	*7.77*
2021	13,537,000	41,124	37,575	27,423	*72.98*	9,890	*26.32*	262	*0.70*	3,364	*8.18*
2022	13,532,000	40,831	37,303	27,292	*73.16*	9,772	*26.20*	239	*0.64*	3,321	*8.13*
2023	13,506,000	43,035	38,998	28,843	*73.96*	9,842	*25.24*	313	*0.80*	3,818	*8.87*
Σ	271,327,000	1,164,566	1,068,267	864,944	***80.97***	196,508	***18.40***	6,815	***0.64***	90,605	***7.78***

Invasive treatments: Sum of all hysterectomies, Myomectomies and fbroid embolizations; None: neither of the three invasive myoma treatments, but other diagnostic and therapeutic procedures (see Table 2)

The absolute numbers of HE decreased from 64,795 to 28,843. This equates to a decline of almost 55.5%. Speaking in relative terms, the percentages of HE compared to the other two therapies decreased from 86.8 to 74%.

The number of ME remained almost constant around an average level of 10,343 ± 401 per year. This corresponds to a relative increase from 12.9% in 2005 to 25.4% in 2023.

When looking at the utilization of UAE, the peak of coded cases was achieved in 2012 with a total of 568 cases in all of Germany. Afterwards, the numbers show a continuous decline with the lowest number of cases in 2022 with only 239 coded procedures. In relative numbers, the uterine artery embolization never even reached 1% of all three treatment strategies.

A slight increase in the number of cases was observed for all three treatment strategies in 2023.

To a small extent, a combination of therapies was coded, mostly ME followed by HE. This probably equates to cases with secondary conversion. In twelve cases UAE was followed by ME during the same hospitalization and in 81 cases it was followed by HE during the same admission.

Ninety thousand, six hundred five hospitalized women received none of the studied therapies (HE, ME or UAE). Table 2 shows the 20 most frequent encoded diagnostic and therapeutic procedures in these cases over the entire period, the Top 3 being hysteroscopy, endometrial biopsy and curettage. During interim analyses, we observed that more than 600 patients received the code for “selective embolization with particles of visceral arteries” (OPS code: 8836 ka). In the context of the chosen primary and secondary diagnoses on admission, these cases were most likely incorrect encoding of UAE procedures. Therefor the authors decided to account these cases as UAE procedures for the final analysis.

**Table 2:** List of the 20 most frequent encoded diagnostic and therapeutic procedures

n	Procedure	OPS codes
44,368	hysteroscopy	1–672
36,145	endometrial biopsy	1–471
20,297	curretage	5–690
11,310	uterine excisions/destructions	5–681
		*including 7399 endometrial ablations (5–681.5)*
9,854	cross-sectional imaging (CT/MRI)	3–8 + 3–2
8,106	laparoscopy	1–694
6,048	esophagogastroduodenoscopy + proctorectocolonoscopy	1–650 + 1–651 + 1–632 + 1–653 + 1–654 + 1–671
5,657	transfusions	8–800 + 8–810 + 8–812.60 + 8–899.7f
5,228	female genital endosonography	3-05d
4,946	adhesiolysis	5–469 + 5–657 + 5–658 + 5–469.2
4,106	biopsies on genital organs	1–472 + 1–571 + 1–570 + 1–573
2,658	surgical excisions on genital organs	5–651 + 5–543 + 5–672 + 5–665 + 5–702 + 5–712 + 5–692
		*1515 salpingoovarectomies, 694 salpingectomies, 164 ovarectomies*
2,373	salpingoovarectomy	5–653 + 5–661 + 5–652
2,194	endoscopic biopsies on gastrointestinal organs	1–440 + 1–444
1,777	fallopian tube insufflation	5–667.1
1,266	excision of ovarian cyst	5–651.92
928	removal of intrauterine device	5–691
585	cystoscopy	1–661
522	vaginoscopy	1–670
353	peritoneal biopsy	1–559.4

*OPS* Operationen- und Prozedurenschlüssel (diagnostic and therapeutic procedure codes), *CT* computed tomography, *MRI* magnetic resonance imaging

## Discussion

This analysis shows the utilization of the three main treatments of symptomatic uterine fibroids in Germany over almost two decades. The numbers of inpatient invasive treatment continuously declined disproportionately to the demographic development primarily due to the impressive decline for the numbers of HE with almost 55.5% over the 19 years. Since there was no relevant increase in the number of cases of alternative invasive treatment strategies and a lack of improvement in purely drug-based treatment this might be interpreted as an indication of a previously high number of unnecessary HE. This may be considered a success, but still the majority of patients (almost ¾) undergo HE. Despite excellent evidence, UAE is not used for treatment of symptomatic uterine fibroids to a relevant extent in Germany and never even reached an annual rate of 1%.

All of the studied therapies (UAE, HE, and ME) are performed as inpatient procedures in Germany and are therefore recorded and billed via the DRG System. Furthermore, the treatments are usually performed and billed in one singular admission. This means that longitudinal patient data regarding repeated inpatient admissions, which are not available in the used DRG statistics, are not relevant for the question considered here. Therefore, the essential weakness of the underlying data and thus of the analysis does not play a significant role in the context of the research question. However, the data does not allow a statement about HE following UAE. The current literature on long-term clinical follow-up after UAE document a secondary HE rate ranging between 9 and 35% [3, 7, 15]. Applying this rate to our dataset, it equates to about 0.07% to 0.28% of all performed HE over the whole timeframe being possibly secondary after UAE.

The slight increase observed in all three treatments in 2023 is most likely a catch-up effect after the end of the SARS-CoV-2 pandemic and probably not a reversal of the observed long-term trend.

The massive underutilization of UAE, although being a proven cost-effective minimally invasive therapy with similar results to HE and ME [1–8], has been described in other countries as well. The 2020 online article on Internationalnews.com about the unequal distribution of UAE and HE reported that on average 2000 UAE are being performed in France per year, while 40,000 patients are being treated by uterine surgeries annually. Even worse, in Spain, only 125–150 UAE per year are in opposition to 50,000 HE. Furthermore, non-European countries show the same trend: in Australia 145 annual UAE are pitted against 30,332 uterine surgeries and El Salvador only had one hospital in 2020 that offered UAE and only 6 UAE are performed on average per year [16]. De Bruijn et al. investigated the development of numbers of UAE after the Dutch national guideline on treatment of uterine fibroids was adjusted to specifically include UAE in 2013 [17]. There was no increase in numbers after the explicit inclusion of UAE in the guidelines. Furthermore, the paper analyzed the recommendation preferences of the gynecologists in all Dutch hospitals and was able to show a grave misconception of risks and effectiveness of UAE. Of all gynecologists in the 69% non-UAE performing hospitals 87% confirmed the false statement, that “after successful UAE, there is a 50% chance of secondary hysterectomy”. If one believes this to be true, it is not surprising that UAE is not being recommended to patients. Frighteningly, at the same time 53% of these gynecologists believe to have sufficient knowledge whereas the rest (47%) claims that there is not enough information available. Furthermore, 54% do not want interventional radiologists to be routinely involved in the counseling process of their patients.

Similar results were shown in a survey study among gynecologists at a gynecologist-specific social media group in the United States of America (USA) about their knowledge and preference concerning UAE [18]. Overall, UAE was offered as treatment to not even 1% of the patients with chronic uterine fibroid symptoms. Free responses given by the gynecologists showed the same grave misconceptions about effectiveness and long-term results as in the Netherlands. 52.1% of the participants believe UAE to be less effective than the other treatments and 68.3% refer patients to UAE rarely or never. However, at the same time, the majority of participants deemed themselves knowledgeable about the risks and effectiveness of UAE.

Among other things, such misjudgements and consecutive miscounselling ultimately lead to a lack of awareness of UAE as an effective treatment alternative among affected women. That could be seen in the results from a poll among women in the USA released by the Society of Interventional Radiology (SIR) back in 2017. It pointed out the missing awareness of UAE of women in general and especially among patients with diagnosed uterine fibroids [19]. Of all women interviewed 59% believed that their gynecologist has discussed all treatment options with them. However, 44% had never heard of UAE and 11% even believed HE to be the only treatment option. Furthermore, only 25% of the patients that did know about UAE had heard about it from their gynecologist. The missing awareness was confirmed by other studies, one of them conducted research about public search trends in Google from 2004 until 2021 [20, 21]. The numbers of searches about UAE showed a peak in the year 2005 but have steadily been declining since then. However, the search numbers for ME rose continuously for the United States and worldwide.

Obviously, the problem of underutilization of UAE is not a German one. The trend is visible all over the world and it seems to be independent from the different health care systems. The main problem seems to be a general distrust and ignorance of gynecologists about the benefits and efficacy of UAE paired with a lack of own active presence and visibility of interventional radiology.

The expected renewal of the German national guideline might strengthen the position of UAE in the treatment of uterine fibroids in the near future. However, as we have seen in the Netherlands this alone will not guarantee a rise in UAE utilization [17]. Furthermore, it appears necessary to actively approach the patients ourselves in multidisciplinary boards and own interventional radiological outpatient clinics to increase the general knowledge and acceptance as well as our visibility. In order to also reach the women affected directly, low-threshold information services such as comprehensible websites, information brochures from specialist societies, as available from the Cardiovascular and Interventional Radiological Society of Europe (CIRSE), and information events like patient webinars should be offered. With great effort and commitment, UAE has been successfully established scientifically and now needs to be further strengthened in the daily clinical practice. It is not too late to give UAE a stronger role and affected women more choices.

## Conclusion

In the last 19 years, the numbers of inpatient invasive treatment of uterine fibroids in Germany continuously declined disproportionately to the demographic development. Despite excellent evidence, UAE is not used for treatment of symptomatic uterine fibroids to a relevant extent in Germany. However, with some effort from interventional radiologists to improve their own visibility and their patients’ awareness of UAE, a turnaround in the current treatments is still possible.

“Die Mühen der Gebirge liegen hinter uns, vor uns liegen die Mühen der Ebenen.“ (Berthold Brecht).

## Data Availability

All data generated or analysed during this study are included in this published article.

## Abbreviations

CIRSE: Cardiovascular and Interventional Radiological Society of Europe
DRGs: Diagnosis Related Groups
HE: Hysterectomy
ICD-10: International Statistical Classification of Diseases and Related Health Problems classification
ME: Myomectomy
OPS: “Operationen- und Prozedurenschlüssel”- diagnostic and therapeutic procedure codes
RDC: Research Data Center
SIR: Society of Interventional Radiology
UAE: Uterine artery embolization
